# Involvement of AP-1 and C/EBPβ in Upregulation of Endothelin B (ET_B_) Receptor Expression in a Rodent Model of Glaucoma

**DOI:** 10.1371/journal.pone.0079183

**Published:** 2013-11-12

**Authors:** Shaoqing He, Alena Z. Minton, Hai-Ying Ma, Dorota L. Stankowska, Xiangle Sun, Raghu R. Krishnamoorthy

**Affiliations:** 1 Department of Cell Biology and Anatomy, University of North Texas Health Science Center, Fort Worth, Texas, United States of America; 2 Department of Pharmacology and Neuroscience, University of North Texas Health Science Center, Fort Worth, Texas, United States of America; 3 Department of Molecular Biology and Immunology, University of North Texas Health Science Center, Fort Worth, Texas, United States of America; University of Florida, United States of America

## Abstract

Previous studies showed that the endothelin B receptor (ET_B_) expression was upregulated and played a key role in neurodegeneration in rodent models of glaucoma. However, the mechanisms underlying upregulation of ET_B_ receptor expression remain largely unknown. Using promoter-reporter assays, the 1258 bp upstream the human ET_B_ promoter region was found to be essential for constitutive expression of ET_B_ receptor gene in human non-pigmented ciliary epithelial cells (HNPE). The −300 to −1 bp and −1258 to −600 bp upstream promoter regions of the ET_B_ receptor appeared to be the key binding regions for transcription factors. In addition, the crucial AP-1 binding site located at −615 to −624 bp upstream promoter was confirmed by luciferase assays and CHIP assays which were performed following overexpression of c-Jun in HNPE cells. Overexpression of either c-Jun or C/EBPβ enhanced the ET_B_ receptor promoter activity, which was reflected in increased mRNA and protein levels of ET_B_ receptor. Furthermore, knock-down of either c-Jun or C/EBPβ in HNPE cells was significantly correlated to decreased mRNA levels of both ET_B_ and ET_A_ receptor. These observations suggest that c-Jun and C/EBPβ are important for regulated expression of the ET_B_ receptor in HNPE cells. In separate experiments, intraocular pressure (IOP) was elevated in one eye of Brown Norway rats while the corresponding contralateral eye served as control. Two weeks of IOP elevation produced increased expression of c-Jun and C/EBPβ in the retinal ganglion cell (RGC) layer from IOP-elevated eyes. The mRNA levels of c-Jun, ET_A_ and ET_B_ receptor were upregulated by 2.2-, 3.1- and 4.4-fold in RGC layers obtained by laser capture microdissection from retinas of eyes with elevated IOP, compared to those from contralateral eyes. Taken together, these data suggest that transcription factor AP-1 plays a key role in elevation of ET_B_ receptor in a rodent model of ocular hypertension.

## Introduction

Glaucoma is an optic neuropathy, characterized by slow degeneration of the optic nerve, cupping of the optic disc, progressive loss of retinal ganglion cells, and visual field deficits that could ultimately result in blindness [1], [2]. Globally, it is estimated that there are over 70 million glaucoma patients [3]. There are well known risk factors associated with glaucoma, including age, race, sex, hypertension, etc. Among these risk factors, increased intraocular pressure (IOP) is the most significantly correlated with glaucoma, especially in primary open angle glaucoma. However, the precise mechanisms by which elevated IOP produces neurodegenerative effects in the retina and optic nerve head are not completely understood. A growing body of evidence suggests that endothelin-1 (ET-1), a 21 amino acid vasoactive peptide, is a contributor to the etiology of glaucoma and is one of the factors increased in response to elevated IOP [2], [4], [5], [6], [7], [8], [9]. ET-1 concentrations have been shown to be elevated in the vitreous humor, aqueous humor and plasma of glaucoma patients and also in some glaucoma models in animals including rat, beagle, etc. [5], [9], [10], [11]. Increased ET-1 concentrations were also found in aqueous humor in the Morrison’s rodent model of ocular hypertension, and ET-1 injected into vitreous induced apoptosis of retinal ganglion cells (RGC) in rats [4], [5], [12].

ET-1 binds to two classes of receptors namely, endothelin A (ET_A_) receptors and endothelin B (ET_B_) receptors, which belong to the rhodopsin superfamily of G protein coupled receptors (GPCRs). ET_A_ and ET_B_ receptors are expressed in many types of cells in the central nervous system (CNS) with ET_B_ receptor being the predominant receptor both in neurons and glia in the CNS [13], [14]. Both receptors are also highly expressed in various ocular tissues including ciliary body, retina and optic nerve head [15], [16], [17]. Upregulation of ET_B_ receptor at the mRNA and protein level was reported in retinas and optic nerves from animal models of glaucoma and also in optic nerve head astrocytic processes in human glaucoma [18], [19], [20]. Our previous study has shown that increased expression of ET_B_ receptor is associated with cell death of RGCs and axon loss in response of elevated IOP, whereas these pathological alterations were greatly attenuated in ET_B_-deficient rats [20]. Molecular mechanisms responsible for regulation of ET_B_ receptor are gaining increased attention, however there are very few studies addressing ET_B_ receptor gene regulation in ocular cells. Using the Promo3 software, our preliminary analysis indicated six binding sites for Activator protein-1 (AP-1) and forty binding sites for CCAAT/enhancer-binding protein β (C/EBPβ) in the promoter of the human ET_B_ receptor gene. Interestingly, increased immunostaining of c-Jun [21] and upregulation of c-Jun and ATF-3 mRNA [22] have been observed in retinas of rats with elevated IOP. In addition, long-term activation of c-Fos and c-Jun in astrocytes was also observed in a monkey model of glaucoma [23]. These observations suggest that AP-1, a transcription factor, may play an important role in gene regulation under glaucomatous conditions. AP-1 is a protein complex comprising of homodimers or heterodimers of basic leucine zipper proteins including Jun, ATF (activating transcription factor), Fos, Maf and JDP, and it is a key transcription factor regulating cell death and survival pathways [24], [25]. CCAAT/enhancer-binding protein (C/EBP) is another member belonging to basic leucine zipper transcription factor family, including C/EBPα, β, γ, δ, ε and ζ. Their cellular roles include regulation of cell cycle, growth and differentiation, and C/EBPs act by binding to the CCAAT motif present in the several gene promoter sequences [26], [27], [28], [29]. Studies in the cardiovascular system have shown that both AP-1 and C/EBPβ are involved in regulating ET_B_ receptor expression in response to external stimuli in vascular smooth muscle cells [30], [31]. However, the functional roles of AP-1 and C/EBPβ in regulating the ET_B_ receptor expression in ocular tissues, especially in glaucoma, are still not clear.

The aim of this study was to investigate the role of transcription factors, AP-1 and C/EBPβ, in upregulation of ET_B_ receptor expression in human non-pigmented ciliary epithelial cells (HNPE) and determine their status in retinal ganglion cells in retinas of rats with elevated IOP.

## Results

### Promoter-reporter Activities of Different ET_B_ Receptor Promoter Constructs and Increased ET_B_ Receptor Promoter Activity Following Overexpression of c-Jun or C/EBPβ in HNPE Cells

**Figure 1:**
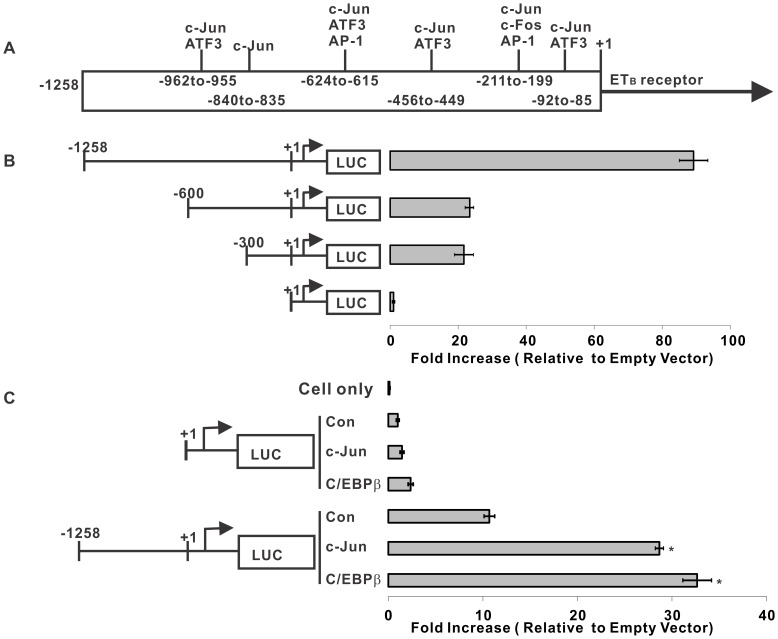
Promoter-reporter activities of promoter truncations and effect of overexpression of c-Jun and C/EBPβ on ET_B_ receptor promoter activity in human non-pigmented ciliary epithelial cells (HNPE). **A.** Diagram of the predicted AP-1 binding sites on human ET_B_ receptor promoter region. Six AP-1 binding sites and forty C/EBPβ sites (not shown in the diagram) were predicted by the Promo-3 software. **B.** Luciferase assay showed promoter activities using different size constructs of the ET_B_ receptor promoter. 1 µg of promoter construct plasmid DNA was transfected into HNPE cells and luciferase activity was measured using a Luciferase assay kit (Promega). The 300 bp and 600 bp upstream promoter constructs yielded 21.7- and 23.4-fold, respectively, increase in luciferase activity, whereas 1258 bp promoter produced an 89.2-fold increase, compared to the empty vector control without a promoter sequence. **C.** Promoter activities of full-length ET_B_ receptor promoter in presence of c-Jun or C/EBPβ co-expression in HNPE cells. 0.5 µg of the human ET_B_ receptor promoter construct was co-transfected with 0.5 µg of either c-Jun or C/EBPβ construct in HNPE cells. The results showed that overexpression of either c-Jun or C/EBPβ boosted the luciferase activity 2.7 and 3.1 fold respectively, compared to that of the ET_B_ receptor promoter construct alone (*p<0.05, One Way ANOVA, Student-Newman-Keuls). Bars represented mean ± SEM, n = 3.

The 1258 bp upstream promoter element of the ET_B_ receptor was analyzed using the software Promo 3 (http://alggen.lsi.upc.es/cgi-bin/promo_v3/promo/promoinit.cgi?dirDB=TF_8.3/). Six AP-1 binding sites (Fig. 1A) and forty C/EBPβ sites (not shown in the diagram) were found on the full length ET_B_ receptor promoter region. Six AP-1 binding sites were located at different regions of ET_B_ receptor promoter, and there were two in −1 to −300 bp, one in −301 to −600 bp and three in −601 to −1258 bp regions of the promoter (Fig. 1A).

Since no retinal ganglion cell line is currently available, the present study was carried out using the transformed human non-pigmented ciliary epithelial (HNPE) cells. The HNPE cell line has been shown to have ET_A_/ET_B_ receptor expression, which was confirmed by ET-1 binding assay in our laboratory [32]. In addition, high efficiency of transfection with plasmid constructs was obtained using this cell line. Promoter-reporter assays using the firefly luciferase gene as a reporter were used to examine the transcriptional activity of different human ET_B_ receptor promoter regions. In presence of 300 bp and 600 bp ET_B_ receptor promoter region (Fig. 1B), there are 21.7- and 23.4-fold increase, respectively, in luciferase activity, compared to that of the empty vector. Furthermore, an 89.2-fold increase in luciferase was detected with 1258 bp promoter region compared to empty vector control, which was a promoter-less vector linked to the luciferase gene. An activity of the positive control vector containing SV40 promoter was about 50–80 fold higher than the full-length ET_B_ receptor promoter vector (not shown in the figures). However, no significant difference was detected in luciferase activity induced by 300 bp- and 600 bp-promoters.

Since the promoter region of ET_B_ receptor (Fig. 1A) was found to have six AP-1 and forty C/EBPβ binding sites, co-expression of either c-Jun or C/EBPβ with luciferase constructs was carried out and luciferase assays were used to determine the effect of overexpression of these transcription factors binding to the ET_B_ receptor promoter. Without the promoter sequence, overexpressed c-Jun or C/EBPβ produced no significant increase in luciferase activity, whereas in the presence of the full length ET_B_ receptor promoter region in the assay, overexpressed c-Jun or C/EBPβ boosted the luciferase activity 2.7- and 3.1-fold respectively compared to that of the full length construct alone (Fig. 1C).

### Promoter Activities of ET_B_ Receptor Promoter Constructs with Mutations at Different AP-1 Binding Sites in HNPE Cells

**Figure 2:**
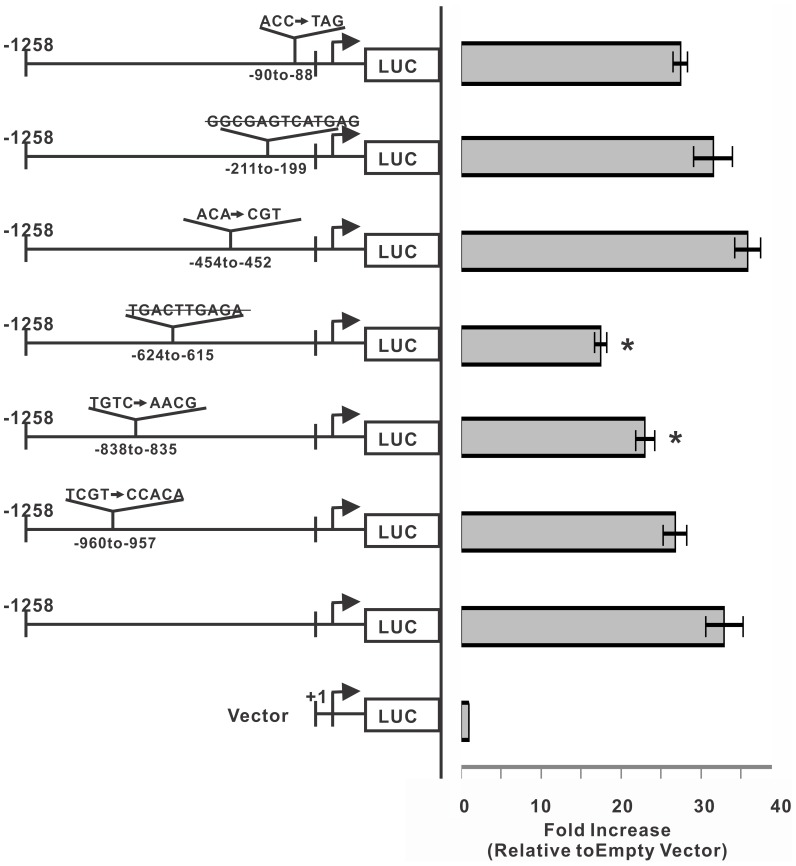
Promoter activities of ET_B_ receptor promoter constructs with mutations in different AP-1 binding sites in HNPE cells. Six AP-1 binding sites were identified in the full-length human ET_B_ receptor promoter region. Corresponding mutations in the different AP-1 binding sites were introduced using site-directed mutagenesis. Luciferase assays were used to determine promoter activities of the full length ET_B_ receptor promoter and six mutant constructs. The deletion at −615 to −624 bp region of promoter abolished the luciferase activity by 45.3% compared to the wild-type ET_B_ receptor promoter (*p<0.05, One Way ANOVA, Student-Newman-Keuls). An apparent decrease in activity was also shown in mutations at −88 to −90 bp, −835 to −838 bp. The results indicated that the −615 to −624 bp AP-1 binding site was very important for ET_B_ receptor promoter activity in HNPE cells. Bars represented mean ± SEM, n = 3.

In order to study the relative contribution of AP-1 binding sites to the ET_B_ receptor promoter activity, six AP-1 binding sites on the full length ET_B_ receptor promoter construct were mutated or deleted using site-directed mutagenesis. The deletion at −615 to −624 bp of ET_B_ receptor promoter region reduced the luciferase activity by 45.3% compared to wild-type promoter (Fig. 2). A modest decrease in activity was also shown in promoter constructs with mutations at −88 to −90 (by 9.1%), −199 to −211 (by 4.6%), and −957 to −960 (by 11.3%) compared to that of the full length promoter; however, the attenuation effects were not statistically significant, compared with full-length promoter construct. Statistical analysis indicated a significant difference (p<0.01) between constructs with mutation in the regions between −615 to −624 bp (by 45.3%), and −835 to −838 bp (by 22.3%), when compared to the full-length ET_B_ receptor promoter construct.

### The −615 to −624 bp Region of the ET_B_ Receptor Promoter is a Crucial AP-1 Binding Site Regulating ET_B_ Receptor Expression in HNPE Cells

**Figure 3:**
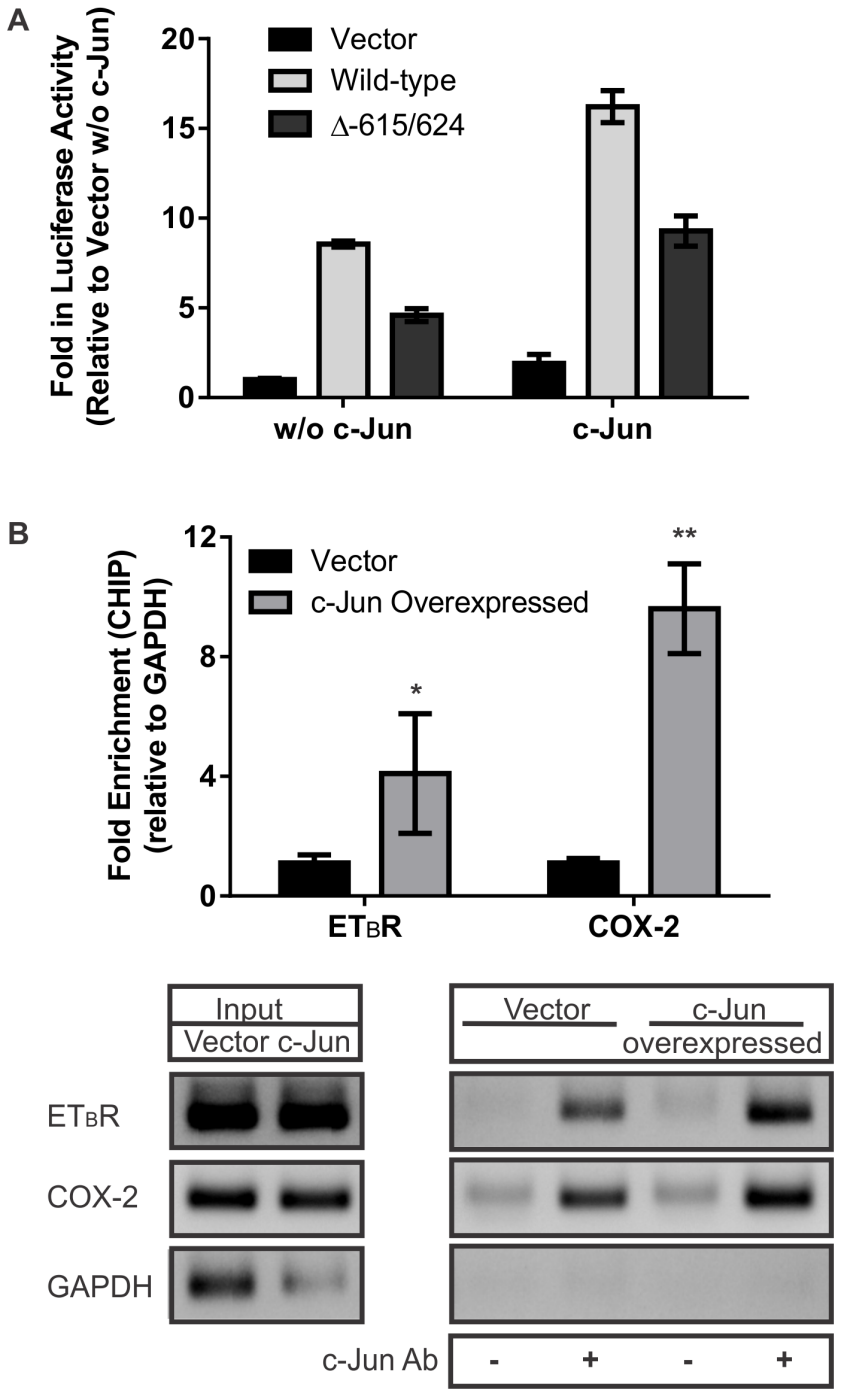
The −615 to −624 bp region of the ET_B_ receptor promoter is the AP-1 binding site in HNPE cells. **A.** 0.5 µg of ET_B_ receptor promoter constructs and 0.5 µg of either the empty vector or c-Jun expressing plasmid DNA were co-transfected into HNPE cells in each well of a 12-well plate. Luciferase assays were performed 24 hours after transfection. In the absence of c-Jun overexpression, luciferase activity obtained from the mutant construct was 54% of the value from wild-type promoter construct. Following c-Jun overexpression, a similar trend of decrease (57% of the wild-type promoter) in promoter activity was observed in the mutant construct. Bars represented mean ± SEM, n = 3. **B.** CHIP assays were performed in HNPE cells, which were transfected with vector control or c-Jun overexpression plasmid DNA. DNA fragments were immunoprecipitated by incubation with or without c-Jun antibody. The data obtained by real-time PCR showed that there was a 4.1 fold increase of AP-1 binding to this specific region of ET_B_ receptor promoter in c-Jun overexpression group compared to vector-transfected control (*p<0.05, student’s *t*-test). The positive control, promoter region of cyclooxygenase-2 (COX-2) was increased 9.6 fold in c-Jun overexpression group (**p<0.005, student’s *t*-test), whereas promoter region of glyceraldehyde 3-phosphate dehydrogenase (*GAPDH)* which served as a negative control showed no appreciable change. Bars represented relative fold increased in data obtained from real-time PCR, n = 4–6. A representative agarose gel shows the amplified DNA from regular PCR at the end of 31 cycles.

Results of luciferase assays using six ET_B_ receptor mutations at different AP-1 binding sites suggest that the binding site at −615 to −624 bp is the most important for ET_B_ receptor transcription. To further address AP-1’s binding and interaction, additional luciferase assays using mutated promoter with coexpression of c-Jun were performed. HNPE cells were co-transfected using either the wild-type full-length ET_B_ receptor promoter construct or full-length ET_B_ receptor promoter containing the deletion at site of −615 to −624 bp, with or without c-Jun overexpression and promoter-reporter assays were carried out. Luciferase activity of the −615 to −624 bp mutant construct was 54% of the value obtained from wild-type promoter construct (Fig. 3A) without c-Jun protein overexpressed. Following c-Jun overexpression, a similar trend of the attenuating effect (57% of the wild-type promoter construct) was observed. There was no statistically significant difference in decrease in promoter activities between these two groups. Experiments were repeated twice in triplicate, the same trend was obtained. Taken together, overexpression of c-Jun didn’t alter the attenuating effect of mutation in this specific binding site to trigger ET_B_ receptor transcription. This suggests that the −615 to −624 bp region is a key AP-1 binding site in ET_B_ receptor promoter.

Furthermore, physical interaction of the DNA in the −615 to −624 bp region of the ET_B_ receptor promoter and transcription factor AP-1 was confirmed by chromatin immunoprecipitation (CHIP) assays. HNPE cells were transfected with either an empty vector or c-Jun expressing plasmid DNA, cross-linked with formaldehyde and sonicated chromatin fragments of 200–800 bp were obtained from the cells. DNA fragments were immunoprecipitated by incubation with or without c-Jun antibody. A pair of PCR primers was designed to amplify a 150 bp fragment of ET_B_ receptor promoter containing AP-1 binding site −615 to −624 bp. The results from real-time PCR showed that there was a 4.1 fold increase of AP-1 binding to this specific region in c-Jun overexpression group compared to vector-transfected control (Fig. 3B). The positive control, promoter region of cyclooxygenase-2 (COX-2), which was upregulated by c-Jun [33], was increased to 9.6 fold in c-Jun overexpression group (Fig. 3B), whereas promoter region of glyceraldehyde 3-phosphate dehydrogenase (*GAPDH)* which served as a negative control showed no appreciable change. Amplification of target fragments was also validated by regular PCR reactions, and PCR products were visualized by a 1.5% agarose gel with SYBR staining (Fig. 3B).

### Regulated ET_A_ and ET_B_ Receptor Expression in Response to Knock-down or Overexpression of c-Jun or C/EBPβ in HNPE Cells

**Figure 4:**
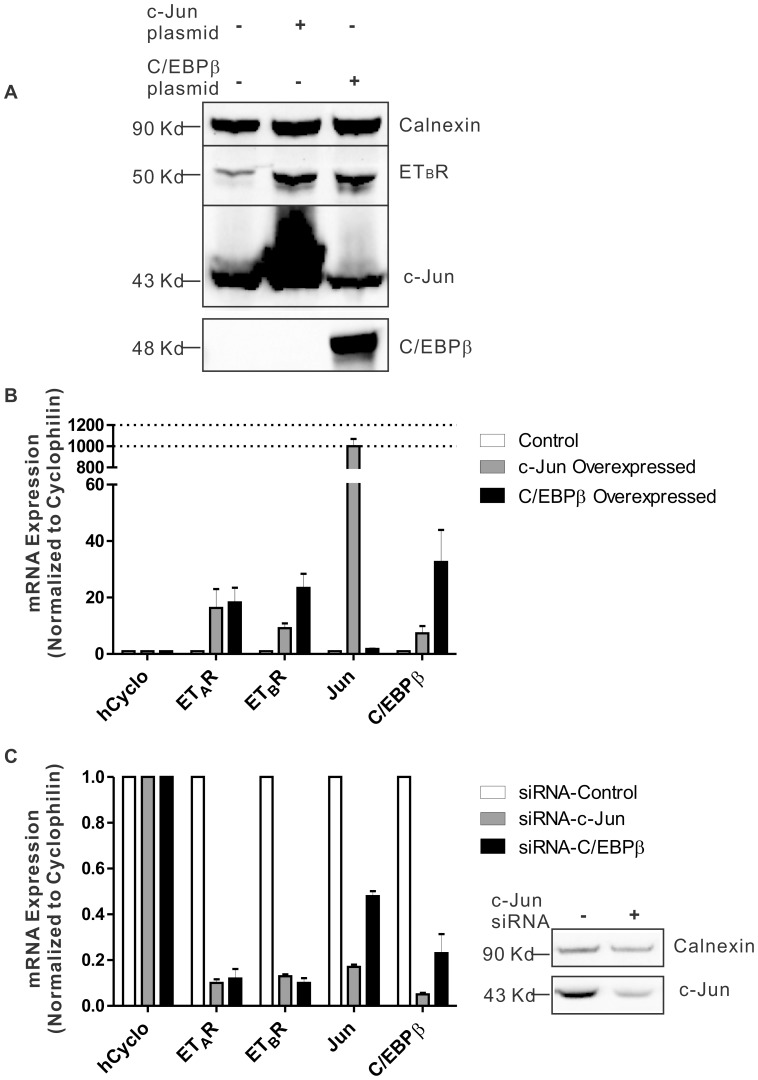
Regulated protein and mRNA levels of ET_A_ receptor, ET_B_ receptor in response to alteration of c-Jun, C/EBPβ expression. **A.** Western blot analysis was used to detect the protein level of ET_B_ receptor in HNPE cells following overexpression of c-Jun or C/EBPβ. ET_B_ receptor protein was upregulated in membrane fractions of HNPE cell when either c-Jun or C/EBPβ was overexpressed. Calnexin, a housekeeping gene, served as a loading control. **B.** Relative mRNA expression of ET_A_ and ET_B_ receptors was analyzed using real-time PCR in HNPE transfected with c-Jun or C/EBPβ plasmid. Overexpression of c-Jun or C/EBPβ triggered an increase of ET_A_ and ET_B_ receptor mRNA level. Results showed as mean ± SEM, n = 3. **C.** siRNAs were employed to knock down c-Jun or C/EBPβ. Total RNA was extracted from HNPE cells transfected with c-Jun or C/EBPβ siRNA and 1 µg of total RNA was transcribed to cDNA. Cyclophilin A served as an internal control for normalization of equal loading of mRNA in the RT-PCR analysis. Knock-down of c-Jun or C/EBPβ significantly reduced the mRNA level of ET_A_ and ET_B_ receptor by more than 5 fold. Knock-down effect in protein level of c-Jun siRNA was also confirmed by Western blot, whereas knock-down effect of C/EBPβ was not detectable.

In order to explore the role of c-Jun and C/EBPβ in the regulation of ET_A_ and ET_B_ receptor, either c-Jun or C/EBPβ was overexpressed by transfection of c-Jun or C/EBPβ constructs into HNPE cells. Following overexpression, protein level of c-Jun and C/EBPβ was detected by immunoblotting and found to be appreciably increased in their corresponding overexpression groups. An enhanced protein level of ET_B_ receptor was detected from membrane fraction of HNPE cells in both c-Jun and C/EBPβ overexpression groups (Fig. 4A). On the other hand, overexpression of c-Jun or C/EBPβ also triggered a 10–23-fold increase of ET_A_ and ET_B_ receptor mRNA level (Fig. 4B). In a different set of experiments, c-Jun or C/EBPβ expression was knocked down by administration of siRNA in HNPE cells. Knockdown of c-Jun or C/EBPβ significantly decreased mRNA level of both ET_A_ and ET_B_ receptor by more than 5 fold (Fig. 4C). Effects of siRNA knock-down of c-Jun or C/EBPβ were confirmed by Western blot and real-time PCR (Fig. 4C). The c-Jun protein level was decreased in c-Jun siRNA knock-down group (Fig. 4C). However, knock-down effect of C/EBPβ was not readily discernible since the endogenous protein level of C/EBPβ was very low and not detectable by Western blot. Interestingly, knock-down of either c-Jun or C/EBPβ abolished the mRNA level of both C/EBPβ and c-Jun. These observations suggest that AP-1 and C/EBPβ are key regulators of ET_B_ receptor expression in HNPE cells.

### mRNA Levels of ET_A_, ET_B_ Receptor and c-Jun were Upregulated in Retinas of Elevated IOP Eyes in Rats

**Figure 5:**
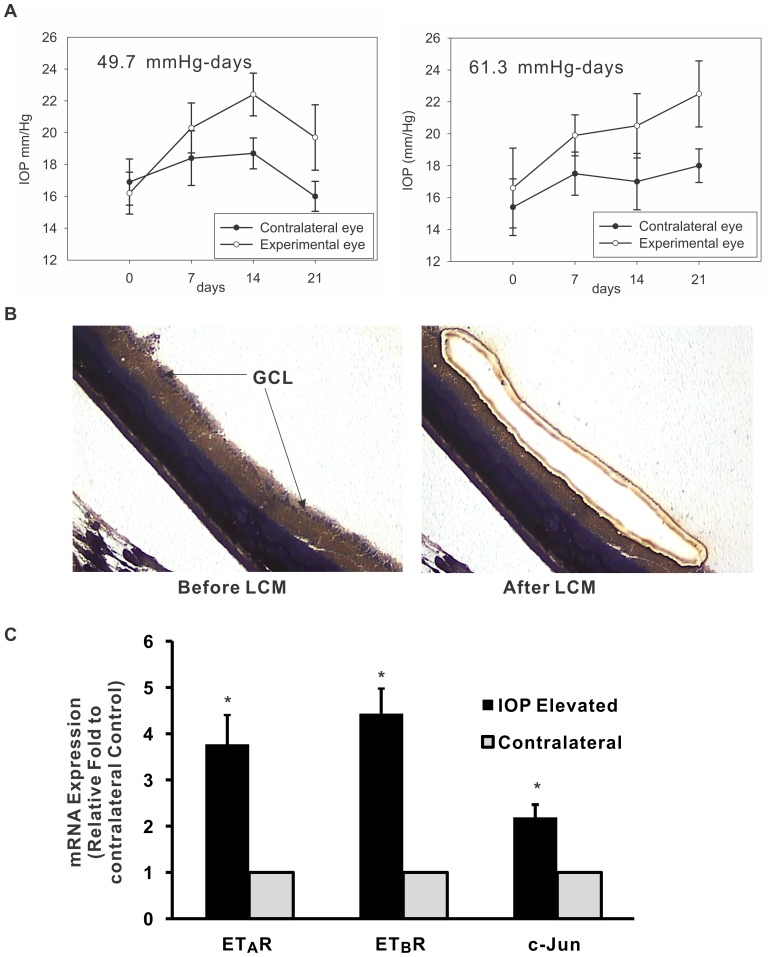
mRNA levels of ET_A_, ET_B_ receptor and c-Jun were upregulated in retinal ganglion cell layer from rats with elevated IOP. **A.** Morrison’s model of glaucoma in rat was developed by injection of hypertonic saline into episcleral veins in the left eye in Brown Norway rats. IOP was monitored twice a week using a Tonolab tonometer. IOP exposure was calculated as the integral product of extent of IOP elevation and the duration for which rats were maintained following IOP elevation and expressed as mm Hg-days. A plot of IOP from treated eye (open circle) and contralateral eye (closed circle) for 3 weeks following surgery indicates the extent of IOP exposure to be 49.7 and 61.3 mmHg days for two rats respectively. The values of IOP obtained are plotted as mean ± SD, n = 10 (10 values was obtained at each time point). **B.** Eyes were enucleated from rats following two-week IOP-elevation in left eyes, while the corresponding right eyes served as contralateral controls. The ganglion cell layers were captured from retina cryosections by Laser Capture Microdissection. **C.** Equal amounts of total RNA extracted from ganglion cell layers were transcribed to cDNA (Pico RNA Extraction, Applied Biosystem). Real-time PCR was employed to detect gene expression using cDNA generated from both IOP elevated and control eyes. Two separate tissue captures and total RNA isolations were carried out for each eye. The analysis showed a 3.1-, 4.4- and 2.2-fold increase in mRNA level of ET_A_, ET_B_ receptor and c-Jun in RGC layer from IOP-elevated eyes, compared to control eyes. Results are shown as mean ± SEM, n = 4. *indicates statistical significance (p<0.01, student’s *t*-test).

To specifically investigate changes in mRNA expression of ET_A_, ET_B_ receptor and c-Jun in response to elevated intraocular pressure (IOP) in a rat glaucoma model, IOP was elevated by injection with hypertonic saline into episcleral veins in the left eye in Brown Norway rats. IOP exposure was calculated as the integral product of the extent of IOP elevation and the duration for which rats were maintained following IOP elevation (which is the difference in areas under the curves of IOP-elevated and contralateral eyes to x-axis) and expressed as mm Hg-days. IOP elevation for 2 weeks in Brown Norway rats typically generated IOP exposures between 45 and 100 mmHg-days, depending upon the extent of IOP elevation (Fig. 5A). Ganglion cell layers (GCLs) from retina sections were captured by Laser Capture Microdissection using freshly generated cryosections from rat eyes (Fig. 5B). Real-time PCR was used to detect changes in level of gene expression in mRNA extracted from captured GCL. There was a 3.1-fold, 4.4-fold and 2.2-fold increase in ET_A_, ET_B_ and c-Jun mRNA levels respectively in IOP elevated eyes compared to contralateral eyes (Fig. 5C).

### Immunostaining of c-Jun and C/EBPβ was Significantly Increased in Retinal Ganglion Cells in Rat Eyes with IOP Elevation for Two Weeks

**Figure 6:**
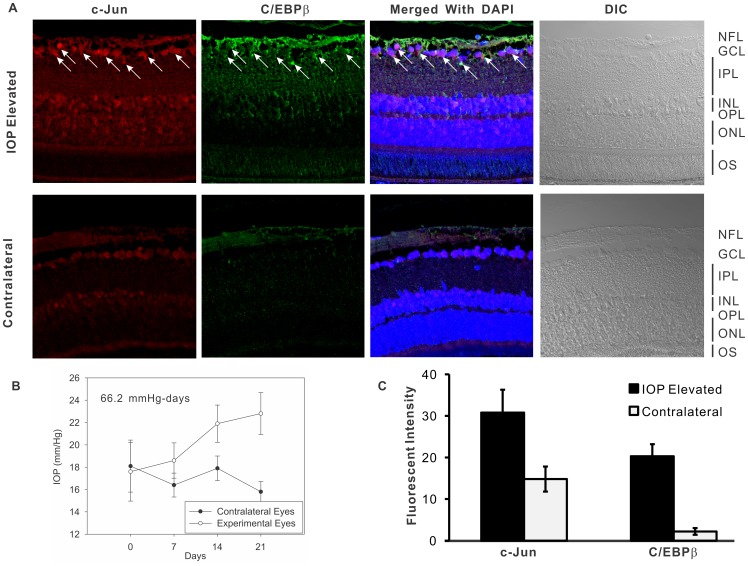
Immunostaining of c-Jun and C/EBPβ was significantly increased in retinal ganglion cells in eyes with IOP elevation for two weeks. The Morrison’s glaucoma model was developed in Brown Norway rats and rats with IOP elevation were maintained for 2 weeks. Paraformaldehyde-fixed retina sections from these rats were stained using specific antibodies to c-Jun and C/EBPβ (Santa Cruz Biotech). Eight to ten images were captured using Z-scan in a Zeiss 510meta confocal microscope from each view and stacked. **A.** Stained RGCs are indicated by white arrows. Red: c-Jun; Green: C/EBPβ; Blue: DAPI. Intense staining of c-Jun and C/EBPβ (indicated by arrows) was detected mainly in RGC layer from retinas of rats and increased staining was observed in retinal sections from IOP elevated rat eyes. **B.** A representative plot from a Brown Norway rat which was subjected to IOP elevation and maintained for 2 weeks. IOP was measured twice a week and values were plotted as mean ± SD (n = 10) for each measurement. The IOP elevation generated 66.2 mm Hg-days of IOP exposure in this rat. **C.** Fluorescent intensity was measured at 10 different regions in the ganglion cell layers using NIH ImageJ. The fluorescent intensity values are shown as mean ± SEM, n = 10.

Since overexpression of c-Jun or C/EBPβ was found to upregulate ET_B_ receptor expression (Figures 1 and 4) immunostaining for c-Jun and C/EBPβ was carried out in retina sections from rats with elevated IOP to determine if these factors are upregulated *in vivo* in the retina following ocular hypertension. Briefly, IOP was elevated in the left eye of six retired breeder Brown Norway rats, while the right eye served as the corresponding contralateral control eye. Following IOP elevation, rats were maintained for 2 weeks, and IOP values obtained and plotted as a function of time. A representative plot of IOP elevation in a Brown Norway rat is shown in Figure 6B which yielded an IOP exposure of 66.2 mm-Hg days. After maintaining rats with elevated IOP for 2 weeks, they were sacrificed. Five micron retina sections were obtained and immunohistochemical staining for c-Jun and C/EBPβ was carried out. Immunohistochemical analyses revealed that there was a significant increase of c-Jun and C/EBPβ immunostaining in IOP-elevated eyes (Fig. 6A). Based upon a quantitative analysis of fluorescent intensity measurements, a 2.1- and 9.1-fold increase in immunostaining for c-Jun and C/EBPβ respectively was obtained in retinas from IOP elevated eyes, compared to contralateral eyes (Fig. 6C). The increased staining was observed primarily in GCL, suggesting that increased levels of c-Jun and C/EBPβ may contribute to upregulation of ET_B_ receptor expression in retinal ganglion cells following elevation of IOP.

## Discussion

Several recent studies suggest that increased expression of the ET_B_ receptor plays a key role in neurodegeneration in glaucoma; however, the gene regulation of ET_B_ receptor is an area that needs further investigation. Knowledge of key regulatory elements mediating ET_B_ receptor upregulation and transcription factors binding to these sites will provide additional tools to block ET_B_ receptor expression, which could be useful to generate neuroprotective approaches. After screening the promoter region of ET_B_ receptor by Promo3 software, six AP-1 binding sites and forty C/EBPβ binding sites were identified. Overexpression of c-Jun or C/EBPβ enhanced the downstream ET_B_ receptor promoter activity by 3 fold compared to the full length promoter control without co-expression of any other transcription factor. This result demonstrated that the AP-1 and C/EBPβ binding sites in the ET_B_ receptor promoter were functional and their cognate transcription factors were able to upregulate gene expression upon binding to these sites. Based on the luciferase activities from truncations, there was no difference in the promoter activity between −300 bp and −600 bp constructs (Fig. 1), which suggested that the AP-1 binding site at the −449 to −456 bp region does not contribute appreciably to regulation of ET_B_ receptor gene expression. Data from the mutation at this site confirmed the conclusion (Fig. 2). Although mutations at three binding sites within −600 to −1258 bp showed differences in their ability to diminish luciferase activities, the AP-1 binding site located at −615 to −624 bp was the most important for ET_B_ receptor promoter activity. This was confirmed by a significant decline in the promoter activity in the construct having a truncation at the −615 to −624 bp site (Fig. 2). Overexpression of c-Jun didn’t alter the attenuating effect of this mutation (Fig. 3A), and physical interaction of AP-1 and the −615 to −624 bp site of ET_B_ receptor promoter was further confirmed by CHIP assays (Fig. 3B). These observations suggest that AP-1 and C/EBPβ are capable of binding to regulatory sites on the ET_B_ receptor promoter region and trigger ET_B_ receptor expression in HNPE cells.

Since AP-1 and C/EBPβ were found to have a positive regulatory effect on the ET_B_ receptor promoter, the role of these two transcription factors in regulation of ET_A_ and ET_B_ receptor mRNA and protein levels was investigated. In the current study, knock-down of either c-Jun or C/EBPβ significantly attenuated the mRNA level of both ET_A_ and ET_B_ receptor. On the other hand, overexpression of c-Jun or C/EBPβ boosted the transcription of ET_A_ and ET_B_ receptor and increased protein level of ET_B_ receptor, suggesting that mRNA expression of both ET_A_ and ET_B_ receptor are regulated mainly by c-Jun or C/EBPβ. Interestingly, upregulation or downregulation of either of these two transcription factors (c-Jun and C/EBPβ) regulated the other in the same manner. It may indicate that there are some interactions between c-Jun and C/EBPβ in HNPE cells. There is evidence to show that the expression of Jun activation-domain binding protein 1 (Jab1), the coactivator of c-Jun, was tightly controlled by binding of C/EBPβ to promoter region of Jab1. Mutations in the C/EBPβ binding site reduced Jab1 promoter activity [34]. Furthermore, the inhibition of c-Jun N-terminal kinases (JNKs) abolished the expression of C/EBPβ and its binding activity [35]. On similar grounds, the lack of C/EBPβ in C/EBPβ^−/−^ mice significantly attenuated ERK1/2, JNKs and their phosphorylated forms [36]. In addition, the direct interaction between Jun and C/EBPβ, which forms the heterodimer, altered the regulatory role of Jun in expression of downstream genes [37], [38]. The detailed mechanisms by which these two transcription factors exert their regulatory roles on each other have not been fully elucidated.

Retinal ganglion cells (RGC) are output neurons located in the innermost layer of the retina, which receive inputs from bipolar cells and fire action potentials which are transmitted to the brain. The percentage of RGCs is less than 1% of total neurons in the retina of human eyes. In order to study gene expression in RGCs *in vivo*, specialized techniques such as Laser Capture Microdissection (LCM) provide us the ability to isolate the retinal ganglion cell layer for a more selective analysis of changes in gene expression in a cell population of interest. In this study, the tissue obtained from LCM still contained other cell types and layers including amacrine cells and nerve fiber layer (Fig. 5B); however, RGCs were the major components in the LCM-captured tissue. In the past two years, Morrison’s group [39], [40] employed the q-PCR and microarray to detect gene expression from LCM captured RGC layers from rat eyes with IOP elevation for five weeks. The authors found an increase in expression of ATF3 (which interacts with members of the c-Jun family) in retinas of rats with elevated IOP. Since AP-1 proteins belong to immediate early gene families, which are activated rapidly in response to a variety of stimuli, we focused on an early time point of IOP elevation (two weeks) and assessed changes in c-Jun and C/EBPβ. The mRNA levels of c-Jun in RGC layer obtained from the 2 week IOP elevated eye were increased to 2.2 fold of control levels, which is consistent with the published results [39], [40], where a 1.4-fold increase of c-Jun was measured in the whole retina and a 5.1-fold increase in RGC layer by real-time PCR from eyes with 5-weeks of IOP elevation. A prominent increase in staining intensity of c-Jun that was observed in IOP-elevated eye could indicate higher protein expression of c-Jun in response to IOP elevation. A similar staining pattern of C/EBPβ was also detected in RGC layer (Fig. 6A). However, the mRNA of C/EBPβ was not detectable with several pairs of different primers. Although the promoter assays in the current study showed that c-Jun and C/EBPβ are upstream regulators to bind the promoter of ET_B_ receptor and activate the transcription of ET_B_ receptor, the direct functional roles of these factors *in vivo* in glaucoma experimental eyes are still unclear. Recently, lack of JNK2/3 signaling due to deficiency of JNK2/3 or Jun in mice has been shown the protective effects from optic nerve crush-induced RGC death [41]. However, the mechanisms that Jun-mediated pathways activate apoptosis of RGC have not been elucidated. It is possible that ET_A_ and ET_B_ receptors are downstream targets of JNK activation and gene expression and some of the protective effects of JNK inactivation may be through attenuation of ET_B_ receptor expression. On the other hand, MAPK, JNK, PKC and PI3K pathways are involved in signaling transduction activated via endothelin receptors, and these pathways subsequently trigger downstream signaling and activate transcriptional factors, such as c-Myc, Elk-1, c-Fos, c-Jun, AP-1, etc. [42], [43], [44], [45], [46]. For instance, ET-1-mediated activation of c-Jun and JNK via endothelin receptors was proved in a variety of cell types and tissues including astrocyte, smooth muscle cells, endothelial cells [4], [33], [47], [48], [49], [50]. Therefore, besides the pathways which regulate the expression of ET_A_ and ET_B_ receptor through AP-1, activated endothelin receptors also modulate the expression of transcription factors in response to the treatment of endothelins and other external stimuli.

There was a more than 4.4-fold increase detected in ET_B_ receptor mRNA level from IOP-elevated eyes. Yang et al., (2007) reported an increase in mRNA level of ET_B_ receptor in whole retinas in a glaucoma rat model using a laser-induced photocoagulation of the trabecular meshwork to elevate IOP [22]. The current study further identified the localization of the increase of ET_B_ receptor gene expression to RGC layer. In addition, a 3.1-fold increase of ET_A_ receptor mRNA was also detected in RGC layer in elevated IOP eyes by real-time PCR. Therefore, it is possible that in addition to ET_B_ receptor, ET_A_ receptor may also be involved in RGC death. Application of bosentan, an antagonist to both ET_A_ and ET_B_ receptor, significantly attenuated glaucomatous alterations in DBA/2J mouse model without changes in blood pressure, IOP elevation and onset of glaucoma [51]. However, the exact role that the ET_A_ receptor plays in the pathogenesis of glaucoma remains to be understood.

In this study, the roles of transcription factors, AP-1 and C/EBPβ, in regulation of ET_B_ receptor was investigated in the HNPE cell line and in an *in vivo* rat model of glaucoma. The 1258 bp upstream promoter region was found to be important for constitutive expression of the human ET_B_ receptor gene. The −615 to −624 bp region is the key binding site of AP-1 in ET_B_ receptor promoter and was found to be crucial for inducible ET_B_ receptor expression. Increased expression of c-Jun and C/EBPβ was associated with upregulation of ET_B_ receptor expression in rat retinas in response to elevated IOP. A comprehensive understanding of the role of AP-1 and C/EBPβ in ET_B_ receptor regulation in glaucoma would help develop molecular tools to control inappropriate ET_B_ receptor expression for neuroprotective approaches in glaucoma.

## Materials and Methods

### HNPE Cells Culture, Transfection of Plasmid DNA and siRNA Knock-down

Human non-pigmented ciliary epithelial (*HNPE*) cells, a kind gift from Dr. Miguel Coca-Prados (Yale University) [52], were propagated using DMEM containing 10% fetal bovine serum in the presence of Penicillin (100 µg/ml) and Streptomycin (100 units/ml). To study the effects of c-Jun or C/EBPβ overexpression, HNPE cells cultured in 100-mm dish were transfected with 10 µg plasmid DNA constructs encoding either c-Jun or C/EBPβ open reading frame using Roche FuGene 6 (Roche Applied Science, Indianapolis, IN). Cells collected 24 hours after transfection were used for RNA isolation/real-time PCR analysis and protein detection. To study the effects of c-Jun or C/EBPβ knock-down, cells cultured in 35 mm dish were transfected with 100 pmole siRNA of c-Jun or C/EBPβ (Santa Cruz Biotechnologies Inc. Santa Cruz, CA) using LipoFectamine 2000 (Invitrogen, Grand Island, NY). Cells collected 24 hours after transfection were used for RNA extraction. Total RNA was extracted using Trizol method (Invitrogen, Grand Island, NY), and RNA quality and quantity were monitored using a nano drop spectrometer. One microgram of total RNA was transcribed to cDNA which served as template for real-time PCR analysis to detect gene expression of ET_A_ receptor, ET_B_ receptor, c-Jun and C/EBPβ. Human cyclophilin A served as an internal control. Primers were used as shown below.

hc-Jun (NM_002228.3):

Forward: 5′- GACCTTCTATGACGATGCCC Reverse: 5′-AGGGTCATGCTCTGTTTCAG

C/EBPβ (NM_005194.2)

Forward: 5′-CCTTTAAATCCATGGAAGTGG Reverse: 5′-GGGCTGAAGTCGATGGC-3′

hET_A_ receptor (NM_001957.3):

Forward: 5′-GCTGGGCGCTGGCCTTTTGA Reverse: 5′-GCGCAGAGGTTGAGGACGGT

hET_B_ receptor (NM_003991):

Forward: 5′-TTCTTGCCTGCGGCCTGTCG Reverse: 5′-TGGCGTTGGAACCCTTGGGC

hCyclophilin A (NM_021130.3):

Forward: 5′-TTCATCTGCACTGCCAAGAC Reverse: 5′-TGGAGTTGTCCACAGTCAGC.

Relative fold-increase in gene expression was calculated by first normalizing to the corresponding cyclophilin A levels and then a ratio to gene expression in the empty vector control was calculated.

### Promoter Constructs, Mutagenesis and Luciferase Assay

The upstream promoter fragments located at 1258 bp, 600 bp and 300 bp from the transcriptional start site of the human ET_B_ receptor promoter were generated by PCR amplification from human genomic DNA and inserted into pGL3 promoter vector carrying the firefly luciferase reporter gene (Promega, Madison, WI). The construct with 1258 bp promoter region served as a full length promoter, the pGL3 with SV40 promoter as the positive control and empty pGL3 without a promoter sequence as a negative control. Six AP-1 binding sites on 1258 bp full length ET_B_ receptor promoter region were identified using the software Promo3 (http://alggen.lsi.upc.es/cgi-bin/promo_v3/promo/promoinit.cgi?dirDB=TF_8.3). Site-directed mutagenesis was employed to mutate each predicted binding site by deletion or change of nucleotides using QuikChange II Site-Directed Mutagenesis Kits (Agilent Technologies, Santa Clara, CA). All the ET_B_ receptor promoter constructs were confirmed by DNA sequencing. The plasmid DNA constructs were transfected into HNPE cells for 24 hours following which cells were disrupted using a lysis buffer (Promega, Madison, WI). The resultant cell lysate (20 µl) was mixed with luciferase reagent (50 µl). Mixture was vortexed briefly and luminescent value was measured immediately in Luminometer (Turner Biosystems, Promega, Madison, WI). Assays were carried out in triplicate and mean values were normalized with corresponding protein amount. The relative fold increase in reporter activity was obtained by calculating the ratio of the activity of the promoter construct to that of the empty vector control.

### Chromatin Immunoprecipitation (ChIP) Assays

ChIP assays were performed to determine if transcription factor AP-1 interacts with the −615 to −624 bp site within the ET_B_ receptor promoter region to provide further evidence to strengthen the data obtained from the luciferase assays. HNPE cells transfected with either the empty vector or the c-Jun overexpressing plasmid DNA for 24 hours were cross-linked by 1% formaldehyde for 10 min at room temperature and subsequently quenched with glycine. Cells were lysed and nuclei were isolated from the transfected cells. The resultant chromatin was resuspended in Lysis Buffer 3 (10 mM Tris-HCl, pH8.0, 100 mM NaCl, 1 mM EDTA, 0.5 mM EGTA, 0.1% Na-deoxycholate, 0.5% N-lauroylsarcosine, and a cocktail of protease inhibitors) and sonicated to obtain DNA fragments at 200–800 bp. The samples were treated with 1% Triton X-100 (final concentration) and subjected to centrifugation at 20,000 g at 4°C for 10 min. The supernatant of samples were incubated overnight at 4°C on rotator with either 4 µg of c-Jun antibody (Santa Cruz Biotechnologies Inc. Santa Cruz, CA) or without antibody which served as the sham control. Following incubation, 25 µl of magnetic A/G beads (Thermo Scientific, Rockford, IL) was added to each reaction and suspension was incubated for 3 hours at 4°C with rotation. The beads were washed four times with RIPA buffer (10 mM Tris-HCl, 0.25 M LiCl, 0.5% NP-40 and 0.5% sodium deoxycholate, pH 7.5) and two times with TE buffer supplemented with 50 mM NaCl. The cross-linking was disrupted by treatment with 10% Chelex-100 by boiling. The samples were treated with RNase A and Proteinase K. The resultant DNA was used as template to analyze AP-1 binding regions within promoter of genes. The following PCR primers were used to assess AP-1 binding to their respective promoters:

ET_B_ receptor promoter (flanking region containing −615 to −624 bp site):

Forward: 5′-GGGTAAAGGAAGGAGCGCG Reverse: 5′- CTACTCCCTGGCTGGCTGAG

COX-2 promoter (positive control: containing AP-1 binding site) [33]:

Forward: 5′- CCCCACCGGGCTTACG Reverse: 5′-GTCGCTAACCGAGAGAACCT

GAPDH promoter (negative control: containing no AP-1 binding site) [53]:

Forward: 5′- ATGGTTGCCACTGGGGATCT Reverse: 5′-TGCCAAAGCCTAGGGGAAGA.

The abundance of promoter sequences that were bound by c-Jun was analyzed by real-time PCR and PCR using 2 µl of DNA as template and SsoAdvanced SYBR Green Supermix (Bio-Rad Laboratories, Hercules, CA). Experiments were repeated at least 4 times in duplicate or triplicate. Fold increase in DNA bound by c-Jun in each experimental group was obtained by subtracting C_T_ values obtained from sham bead control and normalized to GAPDH negative control. The results were confirmed by regular PCR and PCR products were subjected by 1.5% agarose gel with SYBR visualization.

### Morrison’s Rat Model of Glaucoma

Wild-type Brown Norway rats (male, retired breeder, 200–300 g; Charles River, Wilmington, MA) were used in experiments. All procedures were carried out in accordance with the ARVO Statement on the Use of Animals in Ophthalmic and Vision Research under the guidelines of the UNTHSC Institutional Animal Care and Use Committee (IACUC). The animal experimental procedures were reviewed and approved by IACUC with Protocol number: 2011/12-51-A05. The rats were anesthetized using an anesthesia cocktail during surgery and sacrificed with overdose of sodium pentobarbital to minimize suffering. Eight Brown Norway rats were anesthetized using a cocktail of ketamine, xylazine and acepromazine, and surgery was performed in the left eye to elevate intraocular pressure using the procedure developed by Dr. John Morrison as described below [54]. In brief, episcleral veins in rat eye were injected with approximately 50 µl of hypertonic (1.8 M) saline which resulted in scarring of the trabecular meshwork, and produced resistance in the outflow pathway thereby producing IOP elevation. IOP was surgically elevated in the left eyes of rats while the corresponding right eyes without any treatment served as contralateral controls in our experiments. IOP elevation was monitored using a Tonolab tonometer (Icare Finland Oy, Espoo, Finland) twice a week. The rats were sacrificed 2 weeks following IOP elevation with overdose of sodium pentobarbital. Eyes from six rats were fixed with 4% paraformaldehyde and retina sections were obtained for immunofluorescent staining. In a separate experiment, eyes from two Brown Norway rats were used for cryosection for laser capture microdissection to prepare samples for mRNA extraction and real-time PCR.

### Immunoflourescent Staining

The Morrison’s method to elevate IOP was carried out in Brown Norway rats and rats were maintained for two weeks following IOP elevation after which they were sacrificed. Rat eyes were fixed in 4% paraformaldehyde in phosphate buffered saline (PBS) for 4 hours. After paraffin embedding, five-micron sagittal sections of eyes were obtained and immunofluorescent staining was carried out. Briefly, slides were de-paraffinized in xylene, re-hydrated using ethanol washes and PBS wash. After permeabilization using sodium citrate (0.1%) and 0.1% Triton X-100, the sections were washed with PBS and non-specific binding was blocked by incubation with 5% bovine serum albumin and 5% normal donkey serum in PBS for 1 hour. The sections were incubated with combinations of primary antibodies of target proteins (c-Jun and C/EBPβ, Santa Cruz Biotechnologies Inc. Santa Cruz, CA) overnight at 4°C, followed by PBS washes and corresponding secondary antibody (donkey anti-mouse Alexa 488 or donkey anti-rabbit 647-conjugate) incubations. Control sections incubated with only secondary antibodies served as background control. The slides were overlaid by coverslips in antifade medium (FluorSave; Calbiochem, La Jolla, CA). Nearly 8–10 images were captured for each view using Zeiss 510meta confocal microscope with Z-scan, obtained images from each Z-scan were stacked. Fluorescent intensities was measured at 10 different regions in the RGC layer using NIH ImageJ and the mean fluorescence intensities were compared between elevated IOP eyes and contralateral eyes.

### Laser Capture Microdissection and RNA/cDNA Sample Preparation

Brown Norway rats were used for IOP elevation in one eye, while the companion eye served as the corresponding contralateral control. After maintaining the rats with elevated IOP for 2 weeks, the rats were sacrificed by an overdose of pentobarbital and rat eyes were enucleated. The eyes were rinsed with PBS and frozen immediately in Optimal Cutting Temperature (OCT) at −80°C. Cryosections (20 µm) were prepared and stained using Hematoxylin and Eosin **(** *H&E* **)** *staining according the instruction of HistoGene Staining Solution (Arcturus, Cat# KIT0415,* Sunnyvale, CA*)*. The ganglion cell layers were captured from retina cryosection by Laser Capture Microdissection system (Arcturus, Molecular Devices, Sunnyvale, CA). Total RNA from RGC layer was extracted using the PicoPure RNA Isolation Kit (Arcturus, Cat# KIT0204) and cDNA was prepared from equal amount of total RNA using iScript Reverse Transcription Kit (BioRad, Cat# 170-8890). Two separate tissue captures and total RNA isolations were performed for each eye. Real-time PCR (Applied Biosystems, Cat# 4312704) was performed using cDNA as template. Amplification of cyclophilin A served as an internal control and ET_A_ receptor, ET_B_ receptor and c-Jun were amplified using following primers.

rat-ET_A_ receptor (NM_012550.2):

Forward: 5′- AGACCGTCTTCTGCTTGGTT Reverse: 5′- TTCACACCGGTTCTTATCCA

rat-ET_B_ receptor (NM_017333.1):

Forward: 5′-TCGCTCTGTATTTGGTGAGC Reverse: 5′- TTGTCGTATCCGTGATCGTT

rat-Cyclophilin A (NM_017101):

Forward : 5′-CAAGACTGAGTGGCTGGATG Reverse: 5′-GGCTTCCACAATGCTCATG

rat-c-Jun (NM_021835.3):

Forward: 5′-CTTGAAAGCGCAAAACTCCG Reverse: 5′-GACTTTCTGTTTAAGCTGTGCC.

Quantitation of fold-increase in gene expression was done by first normalizing to the internal control, cyclophilin A, and the ratio of gene expression between the elevated IOP eye and the contralateral eye were plotted as histograms.
